# R1398 is the GTP-γ-phosphate sensor that drives the ROC G-domain switching mechanism unique to Parkinson’s disease-associated LRRK2

**DOI:** 10.21203/rs.3.rs-10121216/v1

**Published:** 2026-07-01

**Authors:** Yangshin Park, Chun-Xiang Wu, Rajnesh K. Yadav, Kayla Tennessen, Neo C. Hoang, Cardea W. Hoang, Xylena Reed, Xiaorong Huang, Mark R. Cookson, R. Jeremy Nichols, Jingling Liao, Quyen Q. Hoang

**Affiliations:** * Department of Biochemistry, Molecular Biology, and Pharmacology, Indiana University School of Medicine, Indianapolis, IN 46202,; † Stark Neurosciences Research Institute, Indiana University School of Medicine, Indianapolis, IN 46202,; ‡ Department of Neurology, Indiana University School of Medicine, Indianapolis, IN 46202,; § Laboratory of Neurogenetics, National Institute on Aging, National Institutes of Health, Bethesda, MD 20892,; ¶ School of Environmental and Biological Engineering, Nanjing University of Science and Technology, Nanjing, People’s Republic of China,; ∥ Department of Structural Biology, St. Jude Children’s Research Hospital, Memphis, TN 38105,; ** Academy of Nutrition and Health, School of Public Health, Wuhan University of Science and Technology, Wuhan 430065, People’s Republic of China

**Keywords:** LRRK2, Parkinson’s disease, ROC, Roco proteins, GTPase, R1398H, X-ray crystallography

## Abstract

Mutations in leucine-rich repeat kinase 2 (LRRK2) are the most common genetic cause of Parkinson’s disease (PD). Strategies that directly inhibit the LRRK2 kinase active site have not demonstrated disease-modifying efficacy in recent clinical testing. A naturally occurring protective variant, R1398H, provides an alternative route for understanding how reduced disease risk may be achieved by tuning the regulatory GTPase domain rather than the kinase domain itself. Here, we combine structural, computational, biochemical, and cell-based analyses to define how R1398H alters the Ras of complex proteins (ROC) G domain of LRRK2. Purified ROC carrying R1398H is folded but resolves as a stable homodimer corresponding to the GDP-bound off state previously defined for wild-type ROC. A 2.0 Å A crystal structure shows unambiguous density for H1398 and reveals close superposition with the GDP-bound wild-type ROC dimer. Molecular dynamics modeling predicts that R1398 engages the γ-phosphate of GTP to stabilize switch-region interactions required for activation, whereas histidine at this position weakens γ-phosphate sensing. Consistent with this model, R1398H reduces GTP hydrolysis, selectively weakens GTP-state stabilization while preserving GDP binding, and decreases Rab29-dependent trans-Golgi recruitment of full-length LRRK2. These findings identify R1398 as a γ-phosphate sensor that couples nucleotide chemistry to ROC conformational switching and suggest a genetics-anchored strategy for stabilizing a protective off-state conformation of LRRK2.

Leucine-rich repeat kinase 2 (LRRK2) is genetically and mechanistically central to Parkinson’s disease (PD). Pathogenic variants in LRRK2 cause autosomal-dominant Parkinsonism and contribute to familial and sporadic PD across multiple populations [1–3]. Because several pathogenic variants increase LRRK2-dependent signaling, including kinase-dependent phosphorylation of Rab substrates, much therapeutic work has focused on kinase inhibition [4, 5]. Brain-penetrant ATP-competitive LRRK2 kinase inhibitors such as BIIB122/DNL151 have advanced into late-stage clinical trials and substantially reduce LRRK2-dependent Rab phosphorylation in patients [6, 7]; however, the Phase 2b LUMA trial of BIIB122 recently failed to slow disease progression in early-stage idiopathic Parkinson’s disease despite confirming >90% peripheral LRRK2 kinase inhibition and approximately 30% reduction in cerebrospinal fluid phospho-Rab10, and the program has been discontinued in idiopathic PD [8]. Chronic LRRK2 kinase inhibition produces lamellar-body accumulation in type II pneumocytes and morphologic kidney changes in preclinical species, mirroring the phenotype of LRRK2 knockout animals and indicating that the catalytic activity of LRRK2 is required for normal homeostasis in peripheral tissues [9, 10]. These constraints, together with the clinical failure of direct kinase inhibition, motivate strategies that selectively dampen pathogenic LRRK2 activation rather than globally suppress kinase activity, ideally guided by mechanisms that human genetics has already shown to be safely tolerated and disease-modifying [11].

LRRK2 is a 2,527-amino-acid multidomain enzyme composed of N-terminal solenoid domains followed by a catalytic core containing a Ras of complex proteins (ROC) GTPase domain, a C-terminal of ROC (COR) domain, and a protein kinase domain. Recent structural studies of full-length LRRK2 and LRRK2 assemblies have shown that the catalytic core is embedded in a network of intramolecular contacts that can hold the kinase domain in an autoinhibited arrangement or promote an activated conformation [12–14]. These observations make the ROC domain a plausible control point for long-range communication between nucleotide binding and kinase activation.

ROC belongs to the Roco family of G proteins and differs from canonical Ras-like small GTPases in ways that have complicated mechanistic interpretation. In Ras, the catalytic glutamine in switch II helps organize the hydrolytic water and transition state. This arginine substitution is not, however, universal across the Roco family. The bacterial Roco from *Chlorobium tepidum*, the canonical biochemical model for the Roco G-domain cycle, retains a glutamine at the equivalent switch II position (Q519) yet still exhibits a nucleotide-dependent dimer–monomer cycle and slow intrinsic hydrolysis [15, 16]. The Q to R substitution in LRRK2 is therefore a lineage-specific feature rather than a Roco-wide solution, sharpening the question of whether R1398 contributes a uniquely LRRK2-specific catalytic or nucleotide-sensing role. Whether R1398 is directly catalytic and how it controls nucleotide-dependent conformational dynamics has remained unresolved.

Work from our group established a stable extended ROC construct, ROC_ext_, and showed that ROC is a bona fide GT-Pase whose oligomeric state and switch-region conformation are regulated by guanine nucleotide binding [17, 18]. GDP favors a dimeric “off” state, whereas GTP favors a monomeric “on” state; pathogenic variants such as R1441H/G/C disrupt this cycle, prolonging the “on” state. More recently, our structural and biochemical analyses of full-length LRRK2 supported a model in which ROC acts as a conformational engine that drives a multistep activation of the kinase domain [19]. These studies raised an important question: can a protective mutation act by pushing ROC in the opposite direction, toward a persistently “off” state?

The naturally occurring LRRK2 R1398H substitution is a variant associated with reduced PD risk and is considered a functional protective variant [20]. Together with the pathogenic R1441 substitutions, R1398H establishes an allelic series within a single ROC G-domain in which closely spaced human variants tune Parkinson’s disease risk in opposite directions, providing a rare opportunity to define the mechanism of protection and pathology at the same regulatory module. A previous cell-based study concluded that R1398H increased LRRK2 GTPase activity and Wnt signaling while reducing GTP binding, leading to the proposal that R1398H decreases the active GTP-bound population [21]. The structural mechanism underlying this phenotype, however, has not been defined, and its relationship to ROC conformational switching is unknown.

Here, we tested the mechanism of R1398H using purified ROC_ext_, X-ray crystallography, molecular dynamics simulations, nucleotide-binding assays, intrinsic GTPase measurements, and full-length LRRK2 GTPase activity and cellular localization assays. The results support a model in which R1398 functions as a γ-phosphate sensor that triggers ROC conversion from a GDP-bound off-state to a GTP-bound on-state. R1398H weakens this sensing interaction, prevents switch II ordering, and stabilizes ROC in a persistently off state.

## Results and Discussion

### Residue R1398 is unique to LRRK2.

**Fig. 1.**
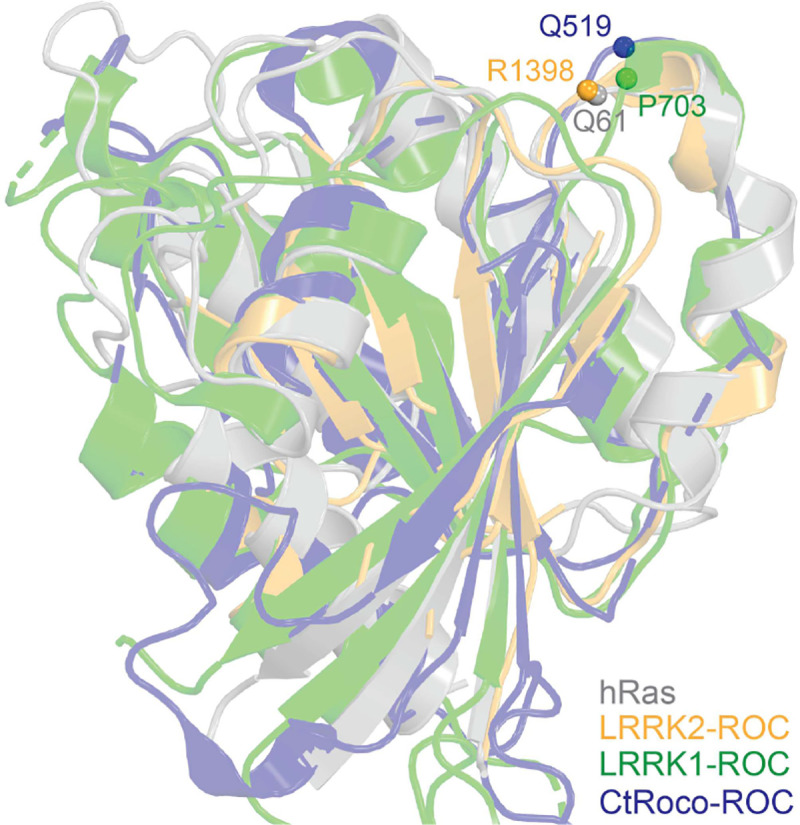
Structures of Roco proteins. Structural alignment of the ROC domain of currently available three-dimensional structures of Roco proteins: human Ras (gray), human LRRK2 (orange), human LRRK1 (green), and *C. tepidum* Roco (blue). The Q61-equivalent positions are shown as spheres.

The mechanism underlying nucleotide-dependent conformational changes and GTP hydrolysis in the ROC domain of LRRK2 remains unclear and is largely inferred from the canonical G protein Ras, in which the catalytic glutamine (Q61) coordinates a water molecule during GTP hydrolysis. However, as noted above, unlike all known classical Ras-family GTPases, LRRK2 carries an arginine at this position rather than the conserved glutamine, a substitution that was identified in the first crystal structure of the LRRK2 ROC domain (PDB: 2ZEJ, 2.0 Å) and described as mechanistically enigmatic [22]. To understand the conservation of the Q61-equivalent position in ROC, we examined all 20 Roco proteins in the UniProt database [23]. Amino acid sequence analysis revealed that 10 Roco proteins retained a glutamine residue at the Q61-equivalent position, whereas the remaining proteins contain diverse noncanonical residues, including hydrophobic residues such as isoleucine, methionine, tyrosine, and proline, with arginine observed only in LRRK2 (Fig. 1, Fig. S1).

These data suggest that the Roco family of G proteins samples diverse mechanisms of GTP sensing and hydrolysis distinct from those of canonical small GTPases, and that LRRK2 is unique among them. The importance of R1398 in LRRK2 is further underscored by the naturally occurring variant R1398H, which confers approximately 20% protection against Parkinson’s disease in Han Chinese and Caucasian populations [20, 24] and independently reduces risk of Crohn’s disease at genome-wide significance [25].

### R1398H stabilizes a dimeric ROC conformation.

**Fig. 2.**
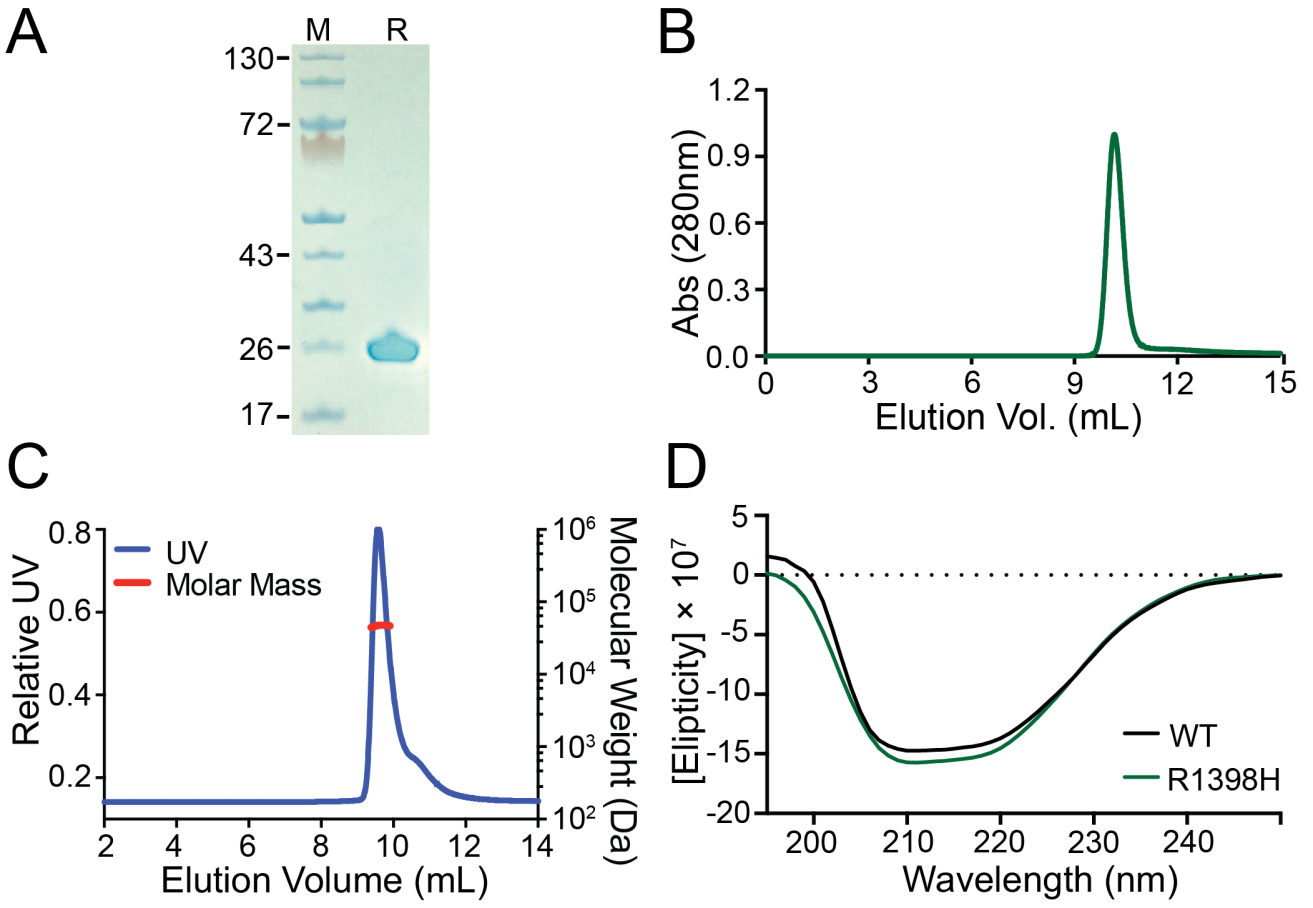
Purification and biophysical analysis of ROC_ext_RH_. (*A*) SDS-PAGE analysis demonstrating the purity of ROC_ext_RH_. (*B*) Size-exclusion chromatography on a Superdex 75 column, showing a single peak consistent with the dimeric conformation. (*C*) SEC-MALS showing a molar mass of 46 ± 2 kDa, consistent with a homodimer. (*D*) CD spectroscopy showing no detectable secondary structure alteration in ROC_ext_RH_ compared with wild-type ROC.

To examine how the R1398H substitution affects ROC structure and activity, we introduced the mutation into ROC_ext_, an extended human LRRK2 ROC construct spanning residues 1329–1520 (ROC_ext_RH_). ROC_ext_RH_ expressed robustly in *Escherichia coli* and purified to near homogeneity by Ni-NTA affinity and size-exclusion chromatography (Fig. 2A).

During size-exclusion chromatography, ROC_ext_RH_ eluted predominantly as a single peak at 10.5 mL, corresponding to the dimeric fraction of wild-type ROC_ext_ rather than the mixed monomer–dimer profile previously observed for wild type (Fig. 2B) [17, 18]. SEC-MALS confirmed an absolute molecular mass of 46 ± 2 kDa, consistent with a homodimer — the theoretical monomeric molecular mass is 23.5 kDa (Fig. 2C).

Circular dichroism spectra of ROC_ext_RH_ overlapped closely with wild-type ROC_ext_, indicating that the R1398H substitution does not detectably perturb the overall secondary structure (Fig. 2D).

The enrichment of ROC_ext_RH_ in the dimeric conformation is notable because the dimeric state corresponds to the GDP-bound “off” conformation defined in our earlier work [17–19]. Thus, before any direct structural interpretation, solution behavior suggested that the R1398H substitution shifts ROC away from the GTP-like “on” state and toward a GDP-like “off” state.

### Crystal structure of ROC_ext_RH_ reveals a GDP-bound “off”-state dimer.

**Fig. 3.**
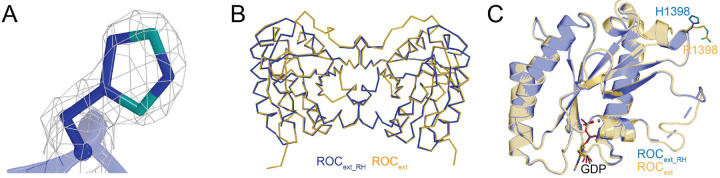
Crystal structure of ROC_ext_RH_. (*A*) Electron-density map (2*F*_o_-*F*_c_) of residue H1398 contoured at 1.0 *σ*. (*B*) Superposition of ROC_ext_RH_ (blue) with GDP-bound wild-type ROC_ext_ (orange); RMSD = 0.25 Å over all C*α* atoms. (*C*) Ribbon representation showing conservation of the nucleotide binding site and GDP (rod-bond model) between ROC_ext_RH_ (blue) and wild-type ROC_ext_ (orange).

To define the structural basis for this shift, we crystallized ROC_ext_RH_ and determined its structure to 2.0-Å resolution (Table S1). Electron density for H1398 was unambiguous, confirming the identity and conformation of the substituted residue (Fig. 3A). The asymmetric unit contains a ROC homodimer with extensive interactions mediated by the switch regions and Interswitch, resembling the GDP-bound dimeric ROC conformation previously described for wild-type ROC_ext_ (Fig. 3B,C) [19].

Superposition of ROC_ext_RH_ with the GDP-bound wild-type ROC_ext_ structure gave an overall RMSD of 0.25 Å over all C*α* atoms. The nucleotide-binding pocket retained a GDP/Mg^2+^ geometry (Fig. 3C). Local inspection showed no evidence of a compensatory structural rearrangement to replace the lost arginine side-chain length and charge.

These structural observations indicate that R1398H does not globally destabilize ROC. Rather, the mutation selectively stabilizes the dimeric GDP-bound conformational state.

### Molecular modeling predicts that R1398 senses the GTP γ-phosphate and orders switch II.

**Fig. 4.**
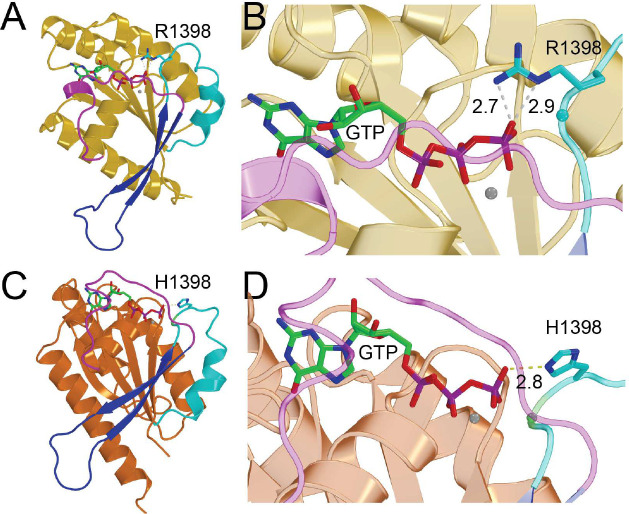
Calculated models of GTP-bound ROC. (*A*) Ribbon representation of ROC_ext_MD_ with bound GTP. R1398 (rod-bond model) interacts directly with the γ-phosphate of GTP. Switch I, switch II, and Interswitch are colored purple, teal, and blue. (*B*) Active-site view showing a bidentate interaction between R1398 and GTP (2.7 and 2.9 Å). (*C*) Ribbon representation of ROC_ext_RH_MD_ with GTP. (*D*) Active-site view showing a single hydrogen bond between H1398 and GTP.

We previously demonstrated that the GTPase activity of ROC_ext_ is coupled with its dimer-to-monomer conformational transition [17]. To examine the GTP-bound state of ROC to investigate the potential molecular basis for the R1398H effect on dimer–monomer dynamics and GTPase activity, we used molecular modeling (because repeated attempts to crystallize GTP-bound ROC were unsuccessful). To build a GTP-bound ROC model, we combined the ROC core from the high-resolution crystal structure of GDP-bound ROC (PDB ID: 6OJF) with the switch-region conformations from a GTP-bound Ras crystal structure (PDB ID: 6Q21), followed by energy minimization, as we recently described [19]. Then, we ran 50 ns of hydrated MD, followed by energy minimization, prior to manual analysis. The resulting wild-type model showed that switch I and switch II were ordered around the nucleotide, consistent with the canonical activation mechanism of small G proteins but adapted to the Roco G-domain architecture (Fig. 4A,C).

Unexpectedly, the wild-type GTP-bound model positioned R1398 within hydrogen-bonding and electrostatic interaction distance of the GTP γ-phosphate (2.7 and 2.9 Å) (Fig. 4B). This positioning places R1398 at the center of the hydrolysis machinery, where it could stabilize the GTP-bound switch II conformation and stabilize the negative charge that develops during phosphate cleavage, analogous to the arginine finger of Ras GAP.

In the R1398H model, the imidazole side chain maintains one hydrogen bond with the γ-phosphate of GTP (Fig. 4C,D). The simulation therefore predicts two linked consequences of R1398H: weakened GTP-state stabilization and impaired ordering of the switch II catalytic arrangement.

This model explains why R1398 occupies a unique position. It is close enough to read the chemical difference between GDP and GTP, yet embedded in switch II so that γ-phosphate recognition can be converted into a larger conformational change by repositioning switch II at the ROC–COR interface. In this view, R1398 is not simply a conventional arginine finger; it is an intramolecular phosphate sensor that couples nucleotide state to LRRK2 conformational switching.

### R1398H weakens GTP-state stabilization and reduces GTP hydrolysis.

The simulations of GTP-bound ROC produced two experimentally testable predictions. First, because R1398 contacts the γ-phosphate, R1398H should have little effect on GDP binding but should weaken GTP binding. Second, the weakened interaction with the γ-phosphate, together with impaired ordering of switch II–helix 3, should reduce GTP hydrolysis. This second prediction could reflect two separable roles for R1398: an upstream conformational role in promoting the GTP-induced dimer-to-monomer transition, and a downstream catalytic role in the hydrolysis reaction itself. We therefore tested nucleotide binding and activity using fluorescence polarization and a GTPase assay.

**Fig. 5.**
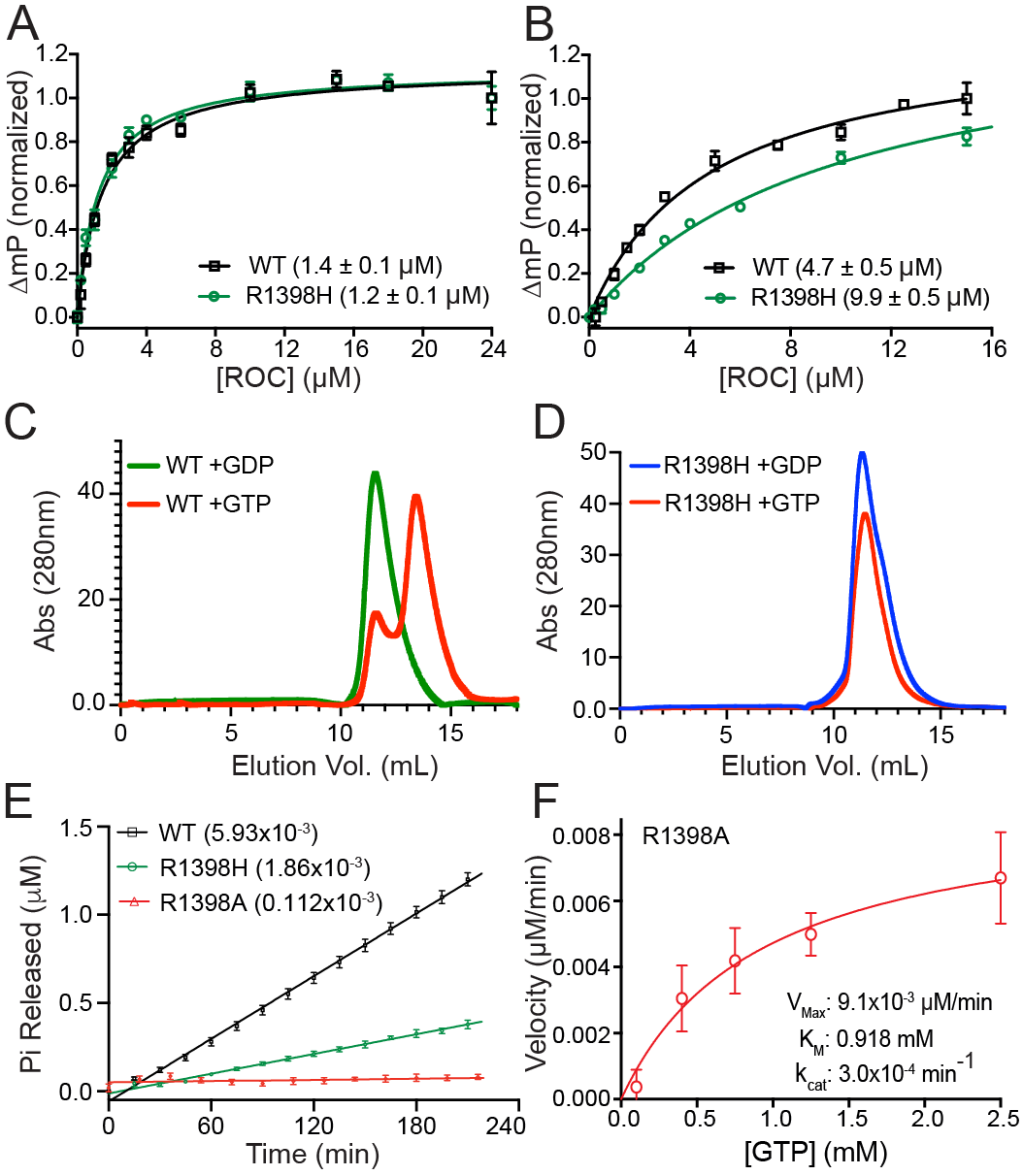
Biochemical properties of R1398H. (*A*) FP assay showing ROC_ext_RH_ (green) binds GDP with essentially the same affinity as wild-type ROC (black). (*B*) FP assay showing ROC_ext_RH_ (green) exhibits twofold lower affinity for GTPγS than wild type (black). (*C*) SEC showing wild-type ROC GDP-bound dimer (green) converts to monomer upon binding GTP (red). (*D*) ROC_ext_RH_ remained dimeric in both GDP- (blue) and GTP-bound (red) states. (*E*) GTPase assay: ROC_ext_RH_ (green) is threefold less active than wild type (black); ROC_ext_RA_ (red) shows nearly undetectable activity. (*F*) Steady-state kinetics of ROC_ext_RA_: twofold increase in *K_m_* and 67-fold reduction in *k*_cat_ relative to wild type.

Fluorescence polarization assays showed that ROC_ext_RH_ binds GDP with an affinity similar to that of WT ROC_ext_, with *K_d_* values of 1.2 ± 0.1 μM and 1.4 ± 0.1 μM, respectively (Fig. 5A). In contrast, its affinity for GTPγS was weakened twofold compared with WT ROC_ext_, with *K_d_* values of 9.9 ± 0.5 μM and 4.7 ± 0.5 μM, respectively (Fig. 5B). These results are consistent with the MD prediction that the loss of the R1398-γ-phosphate interaction selectively weakens the GTP-bound state while having little effect on GDP binding. Consistent with this interpretation, ROC_ext_RH_ lost the ability to undergo GTP-induced dimer-to-monomer conformational change observed for WT ROC_ext_ in the size-exclusion chromatography assay we previously described (Fig. 5C, D) [18]. These data indicate that the R1398-γ-phosphate interaction is required for GTP to drive ROC from the dimeric ?off? state toward the monomeric “on” state.

We next tested whether the “off”-state stabilization of ROC_ext_RH_ affects the GTPase activity of ROC. Intrinsic GTP hydrolysis was measured using the GTPase assay we established for ROC_ext_ [17–19]. In contrast to the literature that R1398H increased GTPase activity in cellular or immunoprecipitated LRRK2 assays [21], purified ROC_ext_RH_ displayed threefold lower GTPase activity than the wild-type ROC_ext_ under identical assay conditions, 1.86×10^−3^ ±1.49×10^−5^ and 5.92 × 10^−3^ ± 3.05 × 10^−5^ μM min^−1^, respectively (Fig. 5E). This difference may reflect the use of purified ROC_ext_ versus immunoprecipitated or cellular full-length LRRK2, different nucleotide loading states, or indirect effects of protein complexes and cellular localization. However, because this assay measures hydrolysis in a system that still requires GTP-induced conformational switching, the reduced activity of ROC_ext_RH_ could not by itself distinguish whether R1398H impairs catalysis directly, indirectly by blocking the conformational transition, or both.

The greater impact of the R1398H substitution on GTPase activity (threefold) than on GTP affinity (twofold) suggests that residue R1398 might also be involved in catalysis. To investigate this, we created the R1398A mutation (ROC_ext_RA_), which lacks the side chain capable of interacting with GTP. We observed that ROC_ext_RA_ is 53-fold less active than WT ROC_ext_ in our GTPase assay, with activities of 0.11 × 10^−3^ and 5.93 × 10^−3^ μM min^−1^, respectively (Fig. 5E). We measured the affinity of GDP and GTP for ROC_ext_RA_. We observed that the GDP affinity remained unchanged (1.18 ± 0.12 μM) (Fig. S2), whereas the GTP affinity decreased slightly (6.42 ± 0.49 μM) compared to WT (4.7 ± 0.5 μM) (Fig. S2). The 53-fold change in GTPase activity cannot be explained by the relatively small 1.4-fold change in GTP affinity, and thus again suggests that residue R1398 may be catalytic. To test this possibility, we carried out steady-state kinetic analysis of ROC_ext_RA_ and found a *K*_*m*_ of 918 ± 575 μM and *k*_cat_ of 3.0 × 10^−4^ ± 1.8 × 10^−4^ min^−1^ (Fig. 5F), which are twofold higher and 67-fold lower than WT, 553 μM and 200 × 10^−4^ min^−1^, respectively [17]. The disproportionate reduction in *k*_cat_ indicates that R1398 contributes to the hydrolytic step itself, not simply to substrate binding.

Together, the R1398H and R1398A data suggest that R1398 has two mechanistically linked functions: it promotes the GTP-dependent conformational change of ROC by engaging the γ-phosphate, and it also contributes directly to efficient GTP hydrolysis. To separate these two functions, we next used a conformational-bypass experiment in which ROC was forced into the monomeric state by a disulfide linkage.

### R1398–GTP interaction triggers switch II translocation at the ROC–COR interface.

The fact that dimer-to-monomer conversion of ROC_ext_ precedes GTP hydrolysis [17], taken together with the large differences between *K*_*m*_ and *K*_*d*_ for GTP observed with our ROC constructs described above, suggests that GTP-dependent conformational change is the rate-limiting step in hydrolysis. This raised an important mechanistic question: does R1398H reduce GTPase activity only because it prevents ROC from reaching the monomeric on state, or does R1398 also contribute directly to catalysis after the monomeric state has formed?

To distinguish between these possibilities, we decoupled dimer–monomer conformational change from GTP hydrolysis by introducing a disulfide bond between switch II (M1409C) and the canonical helix 3 (R1441C), which stabilizes the monomeric conformation [19] and thus bypasses the dimer to monomer conversion step. If R1398 functioned only as a γ-phosphate-dependent conformational trigger, then forcing ROC into the monomeric state should largely eliminate the effect of R1398H on GTP hydrolysis. Instead, R1398H still reduced GTPase activity in the disulfide-stabilized monomeric background.

**Fig. 6.**
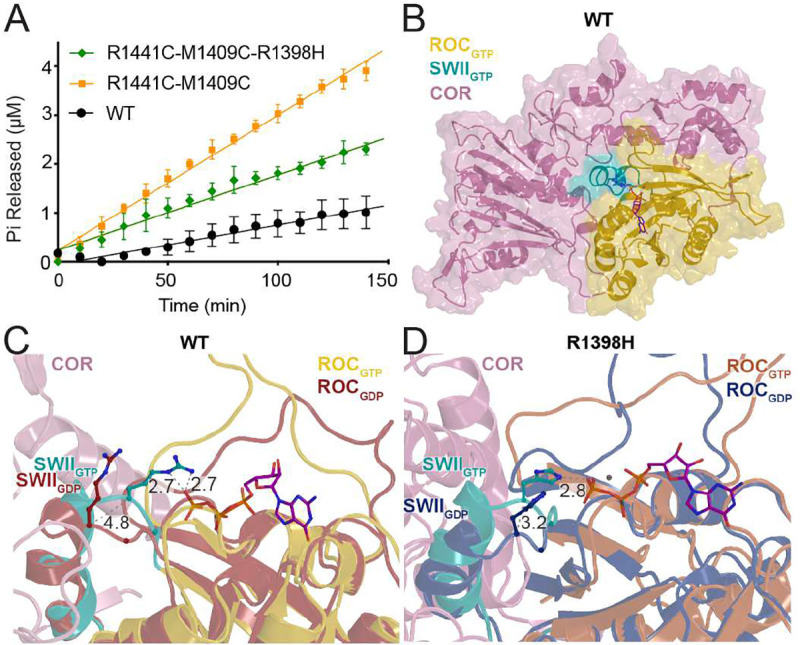
Structural and biochemical properties of R1398. (*A*) GTPase assay: ROC_ext_SS_ (orange) is ~3.5-fold more active than wild type (black); ROC_ext_SS_RH_ (green) is ~1.9-fold more active than wild type. (*B*) MD model of GTP-bound ROC–COR showing switch II (teal) at the ROC (orange)–COR (purple) interface. (*C*) Superposition of GDP-bound (brown) and GTP-bound (orange) active sites: R1398 engages the γ-phosphate with a 4.8-Å switch-II displacement. (D) Superposition of GDP-bound R1398H (blue) and GTP-bound (brown): H1398 engages the γ-phosphate with a 3.2-Å switch-II displacement.

In contrast to Fig. 5E above, where ROC_ext_RH_ showed lower GTPase activity than WT, the monomer-stabilized double mutant, R1441C and M1409C (ROC_ext_SS_RH_), is 1.9-fold more active than WT and 1.9-fold less active than the monomer-stabilized WT R1398 (ROC_ext_SS_), with rates of 0.015, 0.008, and 0.028 μM min^−1^, respectively (Fig. 6A). Thus, enforcing the monomeric “on”-like state partially bypasses the conformational defect of R1398H, but it does not restore WT monomer-stabilized activity. This residual defect demonstrates that R1398 has a direct catalytic role in addition to its role in driving nucleotide-dependent conformational switching.

To investigate whether R1398 can engage GTP within the ROC–COR architecture of full-length LRRK2, and to identify the associated structural changes, we again used molecular modeling, as high resolution structures of GTP-bound LRRK2 are not available. We built a starting model of GDP-bound WT ROC–COR didomain construct by grafting the coordinates of a 1.6-Å crystal structure of ROC_ext_ (PDB ID: 6OJF) onto a 3.7-Å cryo-EM COR domain (PDB ID: 8F09), followed by energy minimization, 100 ns MD run using NAMD [27] and finally energy minimization (ROC-COR_MD_GDP_). We then built the GTP-bound model (ROC-COR_MD_GTP_) by replacing GDP in ROC-COR_MD_GDP_ with GTP, followed by energy minimization and 100 ns MD simulations to accommodate any necessary structural rearrangements. Representative frames with the minimum distance between R1398 and GTP (NH2-OG3) were energy-minimized.

Examination of the GTP-bound MD model showed that residue R1398 had moved from its initial GDP-associated position outside the active site to within bidentate contact distance (2.7 Å) of the γ-phosphate of GTP in the active site (Fig. 6B,C), consistent with our earlier simulations of ROC alone. Least-squares superposition of the GDP- and GTP-bound MD models showed overall conservation of the ROC–COR architecture while revealing localized structural changes in the switch regions. Notably, in the GTP-bound model, switch II, which lies at the interface between ROC and COR, is shifted 4.8 Å toward the GTPase active site and away from the COR domain relative to the GDP-bound model (Fig. 6B,C). These results are consistent with a model in which R1398 engagement with the γ-phosphate of GTP drives switch II toward the GTPase active site, thereby promoting its displacement from the COR domain.

To gain insight into how the R1398H substitution may affect the R1398-GTP-driven switch II movement described above, we carried out the same MD simulation using an R1398H variant model (ROC_MD_RH_GTP_). H1398 in ROC_MD_RH_GTP_ moved from its initial GDP-associated position to within contact distance (2.8 Å) with the γ-phosphate of GTP in the active site, accompanied by a 3.2 Å displacement of switch II (Fig. 6D).

Taken together, these data suggest that residue R1398 senses GTP and drives the translocation of switch II, leading to a structural rearrangement between ROC and COR that culminates in the activated LRRK2 conformation that we and others have recently reported [14, 19]. Moreover, the R1398H substitution appears to weaken the R1398–GTP driving force, thereby stabilizing LRRK2 in a more persistent GTPase “off” state and a kinase-autoinhibited conformation.

### R1398H activity in ROC_ext_ reflects full-length LRRK2.

**Fig. 7.**
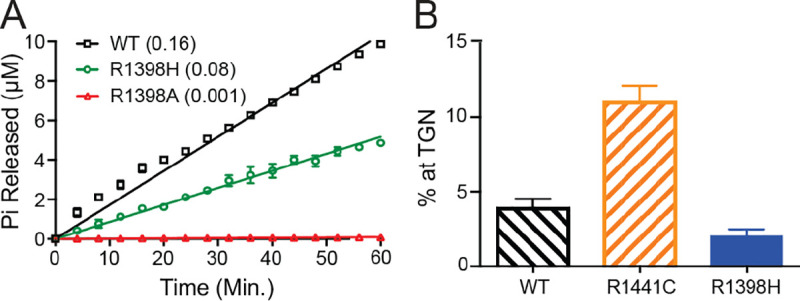
Activity of full-length LRRK2. (**A**) GTPase assay showing that full-length LRRK2_R1398H_ (green) exhibits twofold lower activity than wild type (black), whereas LRRK2_R1398A_ (red) showed nearly undetectable activity. (**B**) Cell-based localization assay: R1441C (orange) causes accumulation of LRRK2 at the TGN relative to wild type (black; p = 0.0001–0.001); R1398H reduces TGN accumulation (p = 0.001–0.01).

To test whether the effect of R1398H on GTPase activity is recapitulated in the full-length LRRK2 context, we introduced the substitution mutation into full-length LRRK2 (LRRK2_R1398H_) and purified it using the two-step purification protocol we recently described [19]. Consistent with ROC_ext_, LRRK2_R1398H_ shows twofold lower GTPase activity than WT (0.085 μM min^−1^ and 0.16 μM min^−1^, respectively) (Fig. 7A), and LRRK2R1398A showed nearly undetectable activity. These data show that the effect of R1398H on ROC_ext_ reflects its effect in full-length LRRK2. This is consistent with our recent report showing the biochemical observations in ROC_ext_ are reflected in full-length LRRK2 and further supports the critical role of R1398 in nucleotide-dependent conformational dynamics and GTP hydrolysis [19].

### The protective mutation opposes pathogenic ROC-dependent subcellular localization.

To test whether the off-state biochemical phenotype is reflected in the behavior of full-length LRRK2 in cells, we examined the Rab29-dependent recruitment of LRRK2 to the trans-Golgi network (TGN), a cellular readout previously used to distinguish conformationally activating ROC mutations [18, 28]. Full-length FLAG-LRRK2 variants were expressed with Myc-Rab29 in HEK293FT cells, and the fraction of cells with LRRK2-positive TGN structures was quantified using TGN46 as a marker.

Consistent with previous observations, the pathogenic R1441C variant increased TGN localization 2.8-fold relative to wild-type LRRK2, with 11.0 ± 0.5% compared with 4.0 ± 0.6% for wild type. In contrast, R1398H reduced TGN localization to 2.0 ± 0.5%, about half of wild type (Fig. 7B).

Although the biological function of LRRK2 remains unclear, the cellular result links the purified ROC mechanism to full-length LRRK2. If ROC activation promotes conformations that favor membrane or TGN recruitment, then R1441C and R1398H should have opposite effects. That is what we observe. R1441C prolongs the active state, whereas R1398H stabilizes ROC in an off state and reduces the TGN recruitment phenotype. These data support a unified model in which ROC nucleotide-state sensing triggers nucleotide-dependent conformational changes that govern both intramolecular activation and intracellular localization of LRRK2. Moreover, the recent BIIB122 clinical trial, in which effective inhibition of LRRK2 kinase activity did not translate into clinical efficacy in idiopathic disease, suggests that kinase activity alone may not define the disease-relevant state. Our data further suggest that LRRK2 conformation and subcellular localization may be critical determinants of disease-associated signaling.

### Conclusion.

**Fig. 8.**
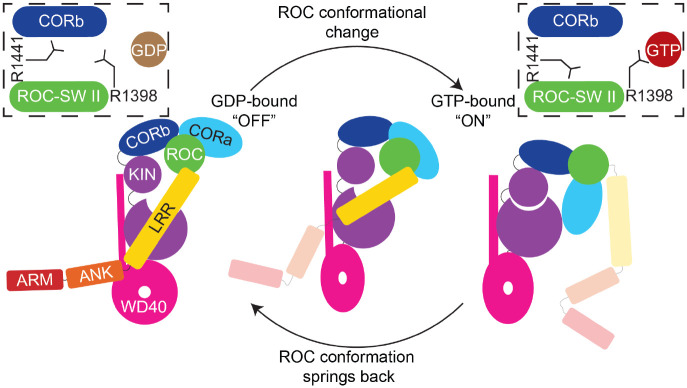
A model of ROC-mediated conformational change and activation of LRRK2. GDP binding stabilizes the “off” autoinhibited conformation of LRRK2, in which R1398 resides outside the active site. In the presence of GTP, R1398 interacts directly with the γ-phosphate of GTP and, in so doing, displaces switch II from the ROC–COR interface, culminating in structural changes that lead to the kinase-active conformation of LRRK2. R1398H (protective) and R1441C/H (pathogenic) variants perturb this cycle in opposite directions: R1398H prevents the GTP-induced “on” transition, whereas R1441C/H prolongs the “on” state.

We recently reported that ROC activates LRRK2 through nucleotide-dependent conformational changes that sequentially detach the N-terminal domains first from the WD40 domain, then the kinase domain, culminating in the exposure of the active site and conformational rearrangement constituting an active kinase geometry [19]. The data presented here identify R1398 as a key γ-phosphate sensor in ROC and support its role in triggering the conformational changes. In wild-type ROC, GTP binding enables R1398 to engage the γ-phosphate, order switch II, and drive the conformational transition from a GDP-bound “off”-state (autoinhibited) toward a GTP-bound “on”-state (activated). GTP hydrolysis or GDP rebinding returns ROC to the “off” state (Fig. 8). R1398H interrupts this cycle by removing the geometry and charge needed for productive γ-phosphate sensing. The result is a GDP-like ROC state with reduced GTP-state stabilization, reduced hydrolysis, and reduced Rab29-dependent TGN localization of full-length LRRK2.

The disulfide-stabilized monomer experiment shows that this defect is not merely a failure to reach the monomeric on state: even when the conformational transition is bypassed, R1398H remains less active than the corresponding R1398-containing monomer, demonstrating a direct catalytic contribution of the R1398 side chain. This mechanism clarifies how a protective genetic variant can act within the same G-domain that harbors pathogenic mutations. Pathogenic ROC mutations (such as R1441C/H/G) impair cycling by prolonging the active state, whereas protective R1398H impairs cycling by preventing activation. The distinction emphasizes that LRRK2 disease biology depends not only on nucleotide binding or hydrolysis rates in isolation, but on how nucleotide chemistry is coupled to conformational state.

Finally, the R1398H mechanism suggests a therapeutic principle. Current LRRK2-directed therapeutics for PD have focused almost exclusively on ATP-competitive type I kinase inhibitors and antisense oligonucleotides that suppress LRRK2 expression, both of which broadly attenuate LRRK2 catalytic output [6, 7]. Although these strategies are clinically tractable, they encounter two recurring challenges. First, chronic global suppression of LRRK2 kinase activity is associated with lysosome-related changes in lung and kidney that mirror Lrrk2 loss-of-function phenotypes [9, 10]. Second, despite being well tolerated and effectively inhibiting LRRK2 kinase activity, the recent investigational drug DNL151/BIIB122 failed to demonstrate therapeutic efficacy [7]. R1398H, in contrast, defines a mechanism that human carriers tolerate across a lifetime while showing reduced PD risk [20, 29], making it a particularly informative template for genetics-anchored target engagement [11]. Our data suggest a complementary pharmacological logic centered on the ROC G-domain rather than the kinase active site: instead of inhibiting LRRK2 solely by targeting the kinase active site, it may be possible to develop modulators that mimic the protective off-state effect of R1398H by weakening the GTP-state switch ordering or stabilizing the GDP-bound ROC conformation.

More broadly, the diversity of residues occupying the Q61-equivalent position across Roco proteins suggests that this family has evolved multiple noncanonical strategies for GTP sensing and hydrolysis, expanding the mechanistic repertoire of the Ras-like G protein superfamily. In LRRK2, the unique catalytic arginine R1398 provides a distinct mechanism for coupling nucleotide state to conformational change and GTP hydrolysis, creating opportunities for mechanism-based therapeutic modulation.

A limitation of this study is that the GTP-bound R1398??-phosphate interaction is inferred from modeling and biochemical perturbation rather than directly visualized in an experimental GTP-bound ROC or ROC?COR structure. In addition, the cellular TGN assay reports localization rather than kinase output. Future structures of GTP- or transition-state-bound ROC?COR and measurements of Rab phosphorylation in R1398H full-length LRRK2 will further test how this protective conformational state modulates downstream signaling.

## Materials and Methods

### Protein expression and purification.

Expression and purification of ROC_ext_ and full-length LRRK2 were carried out as we previously described. Briey, ROC_ext_, consisting of residues 1329–1520, was expressed from Rosetta2 (DE3) E. coli (Novagen) by inducing with 0.5 mM isopropyl-β-D-thiogalactopyranoside (IPTG) for 16 h at 20°C. Cells were harvested by centrifugation and lysed by sonication in a buffer containing 30 mM HEPES (pH 7.4), 250 mM NaCl, 10 mM MgCl_2_, 10 mM glycine, 20 mM imidazole, 10 μM GDP, and 10% (v/v) glycerol. Cell debris was cleared by ultracentrifugation at 140,000 *g* (35,000 rpm, Beckman 45 Ti rotor). The supernatant was incubated with Ni-NTA agarose (Invitrogen) for 2 h at 4°C, then washed with lysis buffer (detailed above) and eluted with buffer containing 30 mM HEPES (pH 7.4), 250 mM NaCl, 10 mM MgCl_2_, 10 mM glycine, 300 mM imidazole, 1 mM DTT, 10 μM GDP, and 10% glycerol. The purified protein was then “polished” by passing through a size-exclusion column (Superdex 200; GE Healthcare) in buffer containing 30 mM HEPES pH 7.4, 150 mM NaCl, 10 mM MgCl_2_, 10 mM glycine, 1 mM DTT, and 10% glycerol. The full-length human LRRK2 constructs were expressed in Hi5 cells for 72 h. Cells were lysed by sonication in a buffer containing 50 mM Tris (pH 7.4), 300 mM NaCl, 10 mM MgCl_2_, 10 mM glycine, 10 μM GDP, 0.006% LMNG, 10% (v/v) glycerol, and protease inhibitor cocktail (Thermo Scientific). Cell debris was cleared by ultracentrifugation at 140,000 *g* (35,000 rpm, Beckman 45 Ti rotor). The supernatant was incubated with anti-FLAG M2 agarose (Sigma) for 3 h at 4°C, then washed with lysis buffer (detailed above) and eluted with buffer containing 50 mM Tris (pH 7.4), 300 mM NaCl, 10 mM MgCl_2_, 10 mM glycine, 0.0002% LMNG, 10% (v/v) glycerol, and 3× FLAG peptide (Fisher Scientific, ApexBio Technology). The eluate was further purified by size-exclusion chromatography (Superose 6 Increase 10/300 GL; GE Healthcare) using the same buffer.

### Size-exclusion chromatography and SEC-MALS.

To determine the absolute molecular weight of ROC_ext_ in solution, we used multiple-angle light scattering. Our experimental setup includes an AKTA FPLC (GE Healthcare Biosciences, Piscataway, NJ) with a silica-based size-exclusion chromatography column (WTC-030S5; Wyatt Technology Corporation, Santa Barbara, CA) as a liquid chromatography unit. Downstream of the column is a refractive index detector (Optilab T-rEX; Wyatt Tech.) followed by a multi-angle light scattering detector (Dawn HeleosII; Wyatt Tech.) for determining protein concentration and particle size, respectively. Each sample injection consisted of ~1 mg of purified ROC_ext_ in buffer containing 30 mM HEPES (pH 7.4), 0.15 M NaCl, 10 mM MgCl_2_, 10 mM glycine, 1 mM DTT, and 10% glycerol. The flow rate was set to 0.4 mL min^−1^, and data were collected at 1-second intervals. Data processing and analysis were performed using ASTRA software (Wyatt Tech.).

### Circular dichroism spectroscopy.

CD spectra were collected on a Biologic Science Instruments MOS450 AF/CD spectrometer with a slit width of 1.0 mm and data acquisition of 1.0 s. The protein samples with concentrations ranging from 0.46 to 0.86 mg mL^−1^ (based on absorbance at 280 nm) were dissolved in a buffer containing 10 mM Tris-HCl (pH 7.4), 150 mM NaCl, 5 mM MgCl_2_, 1 mM DTT, and 5% glycerol.

### Crystallization, data collection, and structure determination.

Purified ROC_ext_RH_ was concentrated to 10 mg mL^−1^ and crystallized by hanging-drop vapor diffusion at room temperature. Crystals were obtained in 100 mM KSCN, 25% PEG MME, and 0.1 M BisTris, pH 6.5. Crystals were cryoprotected in mother liquor supplemented with 10% glycerol and flash-cooled in liquid nitrogen. Diffraction data were collected at 100 K on a Pilatus3 6M detector (DECTRIS, Switzerland) and processed with the program HKL2000 [30]. The structure was solved by molecular replacement using the ROC_ext_ structure (PDB ID: 6OJF) as the search model and refined with PHENIX and Coot [31, 32]. Model quality was assessed with MolProbity [33]. Coordinates and structure factors have been deposited in the Protein Data Bank under accession code PDB ID: 6XAF.

### Molecular modeling and molecular dynamics simulations.

Homology models of the monomeric ROC domain in GTP-bound conformation were built based on the structures of GDP-bound ROC_ext_ and GTP-bound Ras (PDB ID: 6OJF and 6Q21) by using the program Modeller 9.19 [34]. Molecular simulation was performed using NAMD [27]. The base ROC–COR model was built from the 3.7-Å cryo-EM structure of full-length LRRK2 (PDB ID: 8F09) and a 1.6-Å crystal structure of ROC_ext_ (PDB ID: 6OJF). The GTP-bound and R1398H variants of ROC–COR were created by substituting GTP for GDP and H1398 for R1398, respectively, followed by energy minimization. For the R1398H model, H1398 was assigned as the neutral HIE tautomer as a conservative protonation state in the absence of direct experimental pKa information. 100-ns solvated molecular dynamics simulations were performed using the CHARMM36M force field in NAMD [27, 35]. The distance between residue 1398 and the guanine nucleotide was monitored with VMD [36]. Distance trajectories from replicate simulations were used to assess the reproducibility of residue R1398–nucleotide engagement, and representative minimum-distance frames were selected for structural visualization. Molecular graphics display and presentation were performed using PyMOL (www.pymol.org).

### Fluorescence polarization nucleotide-binding assays.

Nucleotide binding affinity of guanine nucleotides BODIPY-FL-GTPγS (100 nM) or BODIPY-FL-GDP (150 nM) (Molecular Probes) was titrated with ROC_ext_ (starting at 0.1 μM) until saturation was reached (15 μM and 10 μM, respectively). Fluorescence polarization signals were read using an EnVision 2102 Multilabel Plate Reader (PerkinElmer, Massachusetts) with excitation at 485 nm and emission at 535 nm. Experiments were performed at 25°C in buffer containing 30 mM HEPES (pH 7.4), 150 mM NaCl, 10 mM MgCl_2_, 10 mM glycine, 4 mM EDTA, 1 mM DTT, and 10% glycerol. Data were analyzed using Prism 10 (GraphPad Software, CA).

### GTPase activity assays.

Intrinsic GTPase activity was measured with the EnzCheck phosphate assay as we previously described [17]. Briefly, ROC_ext_ (30 μM) was incubated with 2 mM GTP in buffer containing 30 mM HEPES (pH 7.4), 150 mM NaCl, 10 mM MgCl_2_, 10 mM glycine, 1 mM DTT, and 10% glycerol at 25°C. Absorbance at 360 nm was recorded every 3 min for 3 h using a microplate reader. The amount of inorganic phosphate released from GTP hydrolysis at each time point was determined by extrapolation using a phosphate standard curve. Data analysis and curve fitting were performed with GraphPad Prism 10.

### Nucleotide-dependent conformational-change assay.

To determine the effect of GDP/GTP cycling on ROC oligomerization, purified ROC_ext_ and ROC_ext_RH_ proteins, 1 mg per sample, were incubated with 16 mM GDP or GTP for 6 h in buffer containing 30 mM HEPES (pH 7.4), 0.15 M NaCl, 10 mM MgCl_2_, 10 mM glycine, 1 mM DTT, and 10% glycerol. Nucleotide incubation was performed in the absence of EDTA. The resulting samples were analyzed by size-exclusion chromatography using a Superdex 75 10/300 GL column (GE Healthcare).

### Cellular localization assay.

The Rab29-dependent localization of LRRK2 to the trans-Golgi network (TGN) was performed as we previously described [18]. Briefly, mutations in ROC were cloned into 3×Flag-LRRK2 using the Quick Change II XL Site-Directed Mutagenesis kit (Agilent Technologies). The resulting plasmids were co-transfected with 2×Myc-RAB29 into HEK293FT cells using Lipofectamine 2000 (ThermoFisher). Proteins were labeled using primary antibodies to FLAG (F1804; Sigma; 1:500), Myc (MCA1929; BioRad; 1:500), and TGN46 (AHP500G; BioRad; 1:1000). AlexaFluor secondary antibodies donkey anti-mouse 488, donkey anti-sheep 568, and donkey anti-rat 647 (ThermoFisher) were used at 1:500. Hoechst 33342 (H3572; ThermoFisher; 1:10000) was used as a nuclear dye. Cells were imaged and overlap of 3×Flag-LRRK2 with TGN46 was quantified using Cellomics ArrayScanVTI HCS Reader (Thermo Scientific) and HCS Studio Version 6.6.0.

## Supplementary Material

Supplementary Files

This is a list of supplementary files associated with this preprint. Click to download.

- Sfigureslegends2.docx

## Data Availability

The atomic coordinates and structure factors for ROC_ext_RH_ have been deposited in the Protein Data Bank under accession code 6XAF. All other data needed to evaluate the conclusions are included in the manuscript and SI Appendix.

## Abbreviations:

LRRK2: leucine-rich repeat kinase 2
ROC: Ras of complex proteins
COR: C-terminal of ROC
PD: Parkinson’s disease
SEC-MALS: size-exclusion chromatography with multi-angle light scattering
FP: fluorescence polarization
TGN: trans-Golgi network
MD: molecular dynamics
